# Comparative Genomics Reveals Extensive Biosynthetic Diversity and Lineage-Specific Metabolic Potential Across *Talaromyces* Species

**DOI:** 10.3390/jof12090690

**Published:** 2026-09-14

**Authors:** Biju Vadakkemukadiyil Chellappan, Hashem Al-Sheikh

**Affiliations:** Department of Biological Sciences, College of Science, King Faisal University, Al-Ahsa 31982, Saudi Arabia; plxha@kfu.edu.sa

**Keywords:** biosynthetic gene clusters, comparative genomics, genome mining, secondary metabolites, *Talaromyces*

## Abstract

The genus *Talaromyces* is an important source of structurally diverse secondary metabolites, yet the conservation and diversification of its biosynthetic potential remain incompletely understood at the genus level. Here, we performed comparative genomic and biosynthetic analyses of 24 *Talaromyces* species to characterize biosynthetic gene cluster (BGC) diversity, gene cluster family (GCF) distribution, and potential relevance to antifungal natural-product discovery. Genome mining identified 1550 BGCs, ranging from 41 to 81 per species, with polyketide synthase (PKS), nonribosomal peptide synthetase (NRPS), terpene, and hybrid PKS–NRPS pathways representing the major biosynthetic classes. The BGCs were grouped into 828 GCFs, of which 567 (68.5%) were species-specific, indicating extensive lineage-level diversification. In contrast, several metabolite-associated biosynthetic systems were conserved across multiple species. Notably, squalestatin S1-associated BGCs occurred in all 24 species but were distributed among 15 distinct GCFs, demonstrating conservation of predicted biosynthetic capacity despite substantial variation in cluster architecture. GCFs associated with characterized antifungal metabolites, including ilicicolin H, leucinostatins, zopfiellin, sordarin, and monorden/monocillins, were also identified. Moreover, 553 GCFs (66.8%) lacked close matches to characterized MIBiG clusters and were classified as chemically unresolved, highlighting a substantial unexplored biosynthetic repertoire. Overall, these findings reveal extensive diversification alongside selective conservation of secondary-metabolite pathways across *Talaromyces* and provide a genomic framework for prioritizing species and BGCs for antifungal natural-product discovery and biocontrol-oriented investigation. These genome-based predictions require metabolomic and functional validation to confirm metabolite production and biological activity.

## 1. Introduction

Fungal diseases are major biological constraints on crop production and food security, causing substantial losses both before and after harvest [1,2]. Disease management remains heavily dependent on synthetic fungicides; however, the emergence of fungicide-resistant pathogen populations, together with concerns regarding environmental persistence, non-target effects, and chemical residues, has intensified the search for sustainable alternatives [3]. Biological control using antagonistic microorganisms and their bioactive metabolites represents a promising complementary strategy for managing phytopathogenic fungi [4]. Filamentous fungi are particularly important sources of structurally and functionally diverse secondary metabolites with antifungal, antibacterial, insecticidal, phytotoxic, and other biological activities [5,6]. Secondary metabolites from nonpathogenic and beneficial fungi can also contribute to agricultural applications, including plant-growth promotion, antagonism against phytopathogens, and biological disease management, as demonstrated for nonpathogenic *Fusarium* and *Trichoderma* species [7,8]. The increasing availability of fungal genome sequences therefore provides opportunities to identify the genetic basis of this chemical diversity and to prioritize biosynthetic pathways with potential applications in sustainable crop protection [9,10].

The biosynthesis of many fungal secondary metabolites is encoded by biosynthetic gene clusters (BGCs), which typically comprise core biosynthetic enzymes together with tailoring, regulatory, and transport genes [11]. Major fungal BGC classes include those encoding polyketide synthases (PKSs), nonribosomal peptide synthetases (NRPSs), terpene synthases, hybrid PKS–NRPS systems, and several less abundant specialized biosynthetic pathways [12]. Genome sequencing has revealed that fungi frequently harbor substantially more BGCs than would be anticipated from their experimentally characterized metabolites, indicating a large reservoir of cryptic or chemically unresolved biosynthetic potential [10]. Genome-mining approaches such as antiSMASH enable systematic identification and classification of these clusters, while comparison with experimentally characterized pathways in MIBiG provides a basis for predicting potential metabolite associations [13]. Nevertheless, sequence similarity to a characterized BGC indicates biosynthetic relatedness rather than direct evidence of metabolite production.

Comparative analysis of BGCs across multiple species provides an additional evolutionary dimension to genome mining. Clustering BGCs into gene cluster families (GCFs) according to their biosynthetic features can distinguish broadly conserved pathways from lineage-restricted biosynthetic systems and reveal diversification of related cluster architectures [14]. Fungal BGCs are evolutionarily dynamic and can diversify through gene gain and loss, duplication, rearrangement, divergence of biosynthetic enzymes, and horizontal transfer [15]. Consequently, closely related fungi may share conserved biosynthetic pathways while simultaneously possessing substantial species- or lineage-specific metabolic capacity. Integrating phylogenomics with BGC and GCF analyses therefore provides a powerful framework for examining how secondary metabolism has diversified during fungal evolution and for identifying poorly characterized pathways that may encode unexplored bioactive chemistry.

The genus *Talaromyces* comprises ecologically diverse fungi with considerable secondary-metabolite diversity and biotechnological potential [16,17]. Several species produce biologically active metabolites or exhibit antagonistic activity against phytopathogenic fungi, supporting their potential relevance to biological control. For example, *Talaromyces rugulosus* has been described as a mycoparasitic fungus and promising biocontrol agent, while genomic and metabolomic studies of other *Talaromyces* species have revealed extensive repertoires of specialized-metabolite BGCs and bioactive compounds [18,19,20,21]. Despite this potential, available studies have largely focused on individual species or isolates. The extent to which biosynthetic pathways are conserved across the genus, restricted to particular evolutionary lineages, or remain unrelated to currently characterized BGCs is therefore incompletely understood. A broader comparative genomic analysis is required to define the conserved and variable components of the *Talaromyces* biosynthetic repertoire and to identify pathways of potential relevance to antifungal activity.

In this study, we performed a comparative genomic analysis of 24 *Talaromyces* species to characterize the diversity, conservation, and distribution of secondary-metabolite biosynthetic pathways across the genus. By integrating phylogenomics, BGC prediction, and gene cluster family analysis, we aimed to identify conserved, lineage-specific, and chemically unresolved biosynthetic systems with potential relevance to natural-product discovery.

## 2. Materials and Methods

### 2.1. Genome and Proteome Datasets

Genome assemblies of 24 *Talaromyces* species were included in the comparative genomic analysis: *T. adpressus*, *T. albobiverticillius*, *T. amestolkiae*, *T. atroroseus*, *T. borbonicus*, *T. funiculosus*, *T. fuscoviridis*, *T. islandicus*, *T. liani*, *T. marneffei*, *T. nanjingensis*, *T. piceae*, *T. pinophilus*, *T. proteolyticus*, *T. purpureogenus*, *T. ruber*, *T. rugulosus*, *T. stipitatus*, *T. stollii*, *T. thailandensis*, *T. trachyspermus*, *T. variabilis*, *T. verruculosus*, and *T. wortmannii*. Genome assemblies and, where available, their corresponding gene annotations and predicted protein sequences were retrieved from publicly available NCBI genome databases. For genome assemblies lacking publicly available protein annotations, protein-coding genes were predicted using AUGUSTUS (version 3.5.0), and the resulting predicted protein sequences were used in subsequent comparative genomic analyses [22]. Assembly accession numbers, genome sizes, numbers of predicted proteins for each species are provided in Table 1.

### 2.2. Comparative Orthologue and Phylogenetic Analyses

Comparative orthologue analysis of the predicted proteomes from the 24 *Talaromyces* species was performed using OrthoFinder (version 3.0) to assess shared and lineage-specific gene repertoires and infer evolutionary relationships [23]. Proteins were clustered into orthologous groups, and orthogroups present in all species were defined as the conserved core set, whereas those restricted to individual species or subsets of species were considered lineage-specific or variably shared. Patterns of orthogroup distribution among species were visualized using an UpSet plot (version 0.9.0) [24]. For phylogenomic analysis, 50 single-copy orthologues shared across all 24 species were selected from the OrthoFinder results. Protein sequences for each orthologue were individually aligned using MEGA (version 11) [25], and poorly aligned or ambiguously aligned regions were removed using trimAl [26]. The curated alignments were subsequently concatenated into a single supermatrix. A maximum-likelihood phylogenetic tree was reconstructed from the concatenated alignment in MEGA 11 (version 11), and branch support was assessed by 100 bootstrap replicates [27].

### 2.3. Prediction and Classification of Biosynthetic Gene Clusters

Biosynthetic gene clusters (BGCs) were identified independently in the 24 *Talaromyces* genomes using antiSMASH (version 8.0) [13]. The proteins encoded within predicted BGCs were extracted and subjected to Pfam domain analysis [28]. Based on the domains, the predicted clusters were classified according to their core biosynthetic machinery, including polyketide synthase (PKS), non-ribosomal peptide synthetase (NRPS), terpene, hybrid PKS–NRPS, and other BGC classes. Clusters containing multiple biosynthetic signatures were classified according to the corresponding antiSMASH annotations. The number and distribution of BGC classes were compared across species to evaluate interspecific differences in secondary-metabolite biosynthetic capacity. Predicted BGCs were additionally compared with experimentally characterized biosynthetic pathways using the KnownClusterBlast/MIBiG-associated annotations provided by antiSMASH [29]. KnownClusterBlast/MIBiG matches meeting a predefined cluster-level similarity threshold (>45% according to the antiSMASH KnownClusterBlast output) were retained as putative metabolite-associated matches. BGCs lacking MIBiG matches above this threshold were designated chemically unresolved. These MIBiG-based assignments were interpreted as evidence of biosynthetic similarity to characterized pathways and not as confirmation of production of the corresponding metabolites.

### 2.4. Gene Cluster Family Analysis

To assess the conservation and diversification of secondary-metabolite biosynthetic potential across *Talaromyces,* BGCs predicted by antiSMASH were grouped into gene cluster families (GCFs) using BiG-SLiCE (version 1.0) [14]. Clustering was based on similarities in biosynthetic domain composition among BGCs. The presence or absence of each GCF was determined across the 24 species to evaluate patterns of cross-species distribution. GCFs detected in a single species were classified as species-specific, whereas those occurring in two or more species were considered shared. Selected BGCs representing broadly conserved, lineage-specific, or metabolite-associated GCFs were comparatively visualized using clinker version 0.28 [30].

## 3. Results

### 3.1. Comparative Orthologue Analysis and Phylogenetic Relationships Among Talaromyces Species

**Figure 1:**
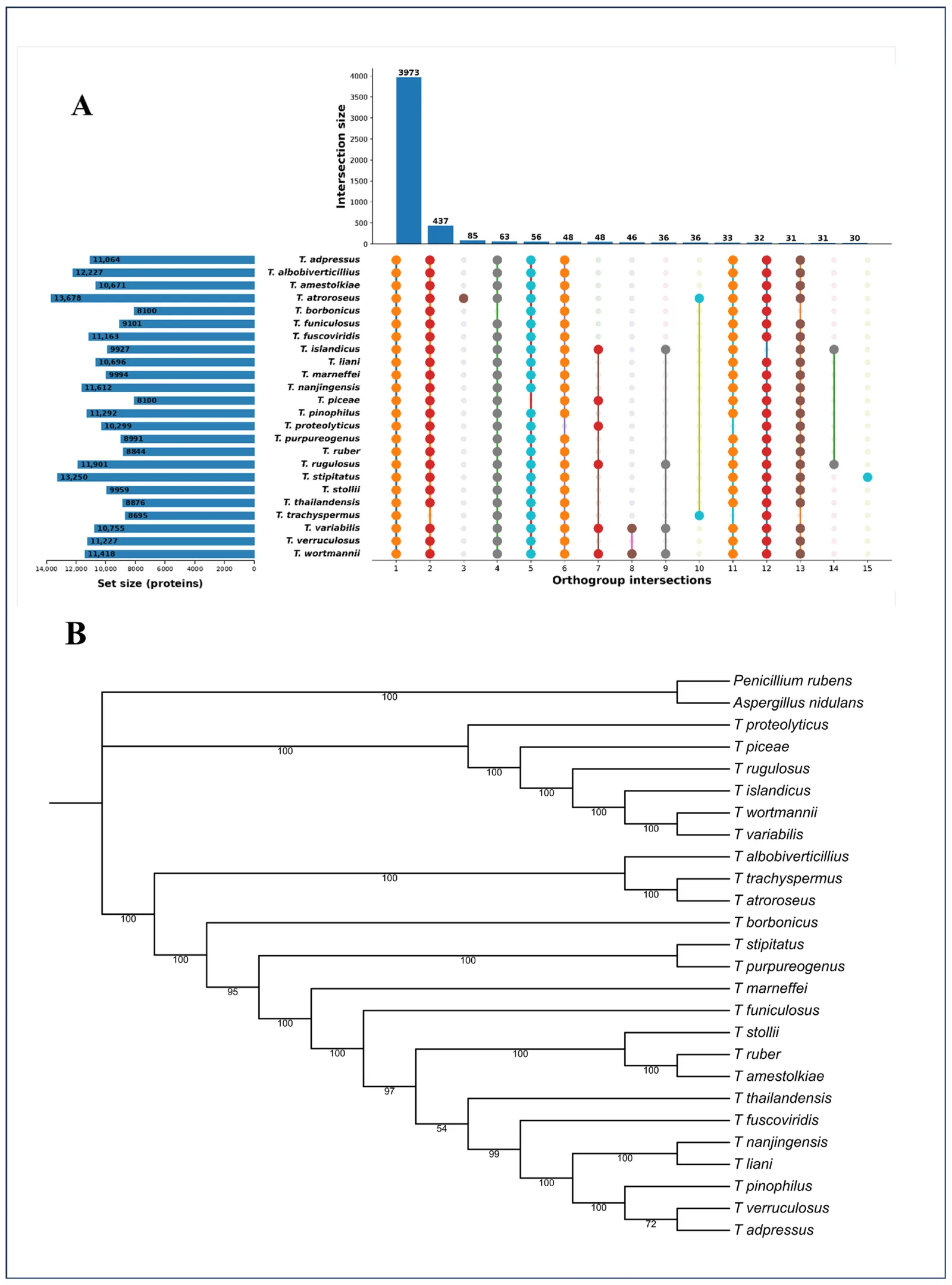
Comparative orthologue distribution and phylogenomic relationships among 24 *Talaromyces* species. (**A**) UpSet plot showing the major intersections of orthogroups across the 24 *Talaromyces* proteomes. Horizontal bars indicate the total protein set size for each species, vertical bars indicate the number of orthogroups represented by each intersection, and connected dots identify the species contributing to individual intersections. (**B**) Maximum-likelihood phylogenomic tree reconstructed from concatenated conserved single-copy orthologues. *Penicillium rubens* and *Aspergillus nidulans* were included as outgroups. Numbers at internal nodes indicate bootstrap support values.

The 24 *Talaromyces* genomes varied in size from 26.6 Mb in *T. piceae* to 42.5 Mb in *T. nanjingensis*, while predicted proteomes ranged from 8100 proteins in *T. borbonicus* and *T. piceae* to 13,678 in *T. atroroseus* (Table 1). OrthoFinder analysis of 251,840 predicted proteins identified 9175 orthogroups, with 248,641 proteins (98.73%) assigned to orthogroups and 3199 (1.27%) remaining unassigned. A total of 3973 orthogroups were present in all 24 *Talaromyces* species, including 1576 single-copy orthogroups. In addition, 4996 orthogroups showed variable distributions across 2–23 species, of which 841 were present in 23 species, whereas 206 orthogroups were species-specific (Figure 1A).

Phylogenomic reconstruction based on the concatenated alignment of 50 selected single-copy orthologues resolved the 24 *Talaromyces* species into distinct lineages, with *Penicillium rubens* and *Aspergillus nidulans*, representatives of related genera outside *Talaromyces* within the order Eurotiales, included as external taxa for rooting the phylogenetic tree (Figure 1B). Most internal nodes showed high bootstrap support. *T. wortmannii* and *T. variabilis* formed a sister group, while *T. liani* and *T. nanjingensis* were recovered as closely related taxa. *T. stipitatus* and *T. purpureogenus* also formed a supported grouping within the reconstructed phylogeny. The complete relationships among the 24 species are shown in Figure 1B.

### 3.2. Genome-Wide Identification of Biosynthetic Gene Clusters

**Figure 2:**
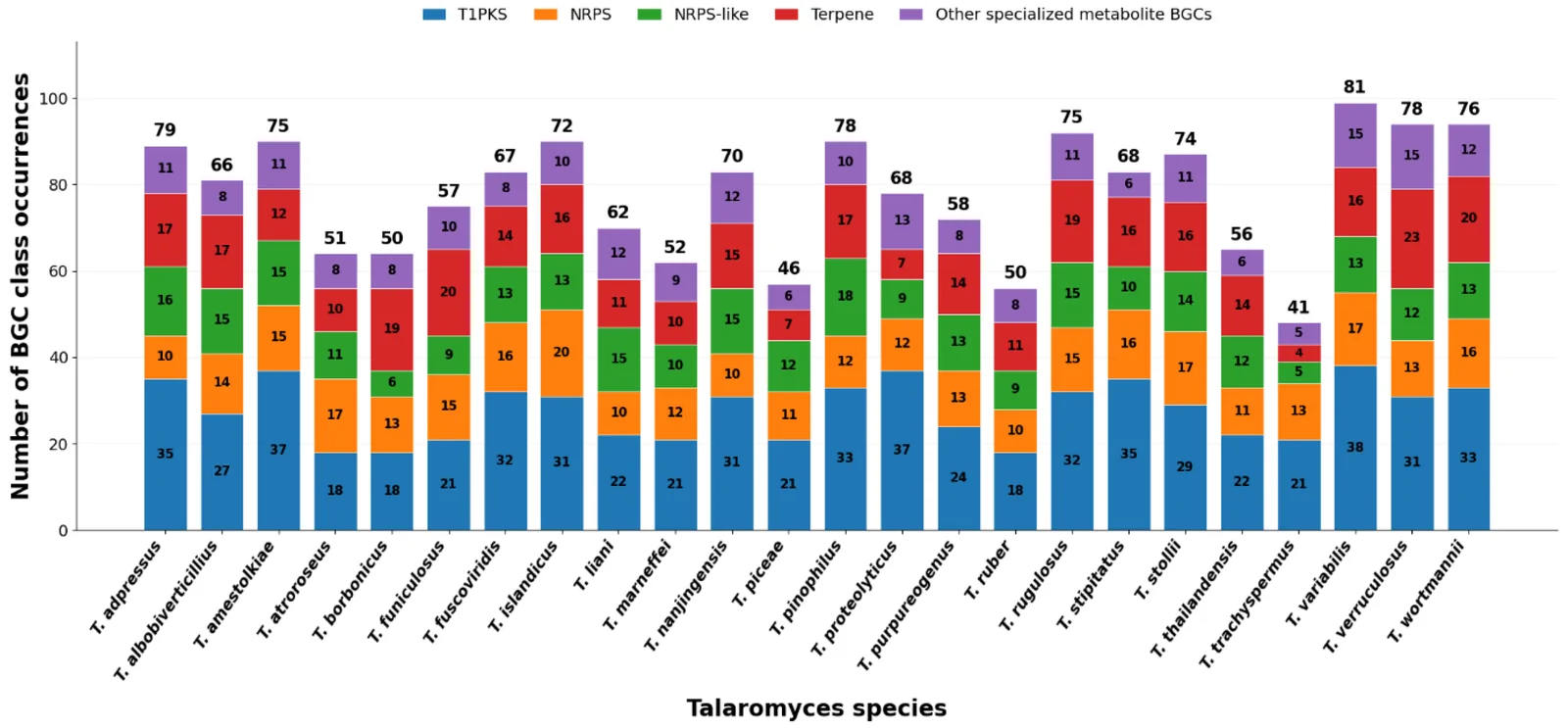
Distribution of major biosynthetic gene cluster classes across 24 *Talaromyces* species. Stacked bar plot showing the abundance and composition of antiSMASH-predicted BGCs in each genome. BGCs are grouped into the major T1PKS, NRPS, NRPS-like, and terpene classes, with less abundant classes combined as other specialized-metabolite BGCs. Numbers within bar segments indicate class-specific counts, and values above the bars indicate the total number of predicted BGCs per species.

Genome mining with antiSMASH identified 1550 biosynthetic gene clusters (BGCs) across the 24 *Talaromyces* genomes (Figure 2), with the number of BGCs per genome ranging from 41 in *T. trachyspermus* to 81 in *T. variabilis*. The predicted clusters encompassed diverse biosynthetic classes, including type I polyketide synthase (T1PKS), nonribosomal peptide synthetase (NRPS), NRPS-like, terpene, hybrid PKS–NRPS, fungal RiPP, siderophore, indole, and other specialized biosynthetic classes. BGC abundance did not scale directly with proteome size. *T. variabilis*, with 10,755 predicted proteins, contained the largest BGC repertoire (81), whereas *T. atroroseus* and *T. stipitatus*, with larger proteomes of 13,678 and 13,250 proteins, respectively, contained fewer BGCs. Conversely, *T. trachyspermus* contained the fewest BGCs despite having a proteome size comparable to several species with larger BGC repertoires.

### 3.3. Comparative Analysis of Polyketide Synthase Genes and PKS-Containing Biosynthetic Gene Clusters

Of the 1550 predicted BGCs, 667 (43.0%) contained at least one polyketide synthase (PKS)-associated component. The number of PKS-containing BGCs ranged from 18 in *T. atroroseus*, *T. borbonicus*, and *T. ruber* to 38 in *T. variabilis* (Figure 2). High numbers were also detected in *T. amestolkiae* and *T. proteolyticus* (37 BGCs each) and in *T. adpressus* and *T. stipitatus* (35 each).

A total of 795 PKS-associated proteins were identified within the 667 PKS-containing regions, including 86 fused PKS–NRPS proteins, which are considered separately in Section 3.5 (Table S1). PKS protein abundance ranged from 20 in *T. ruber* to 45 in *T. amestolkiae* and *T. variabilis*. Other species with relatively high PKS protein numbers included *T. proteolyticus* (43), *T. adpressus* (42), and *T. rugulosus* (41).

**Figure 3:**
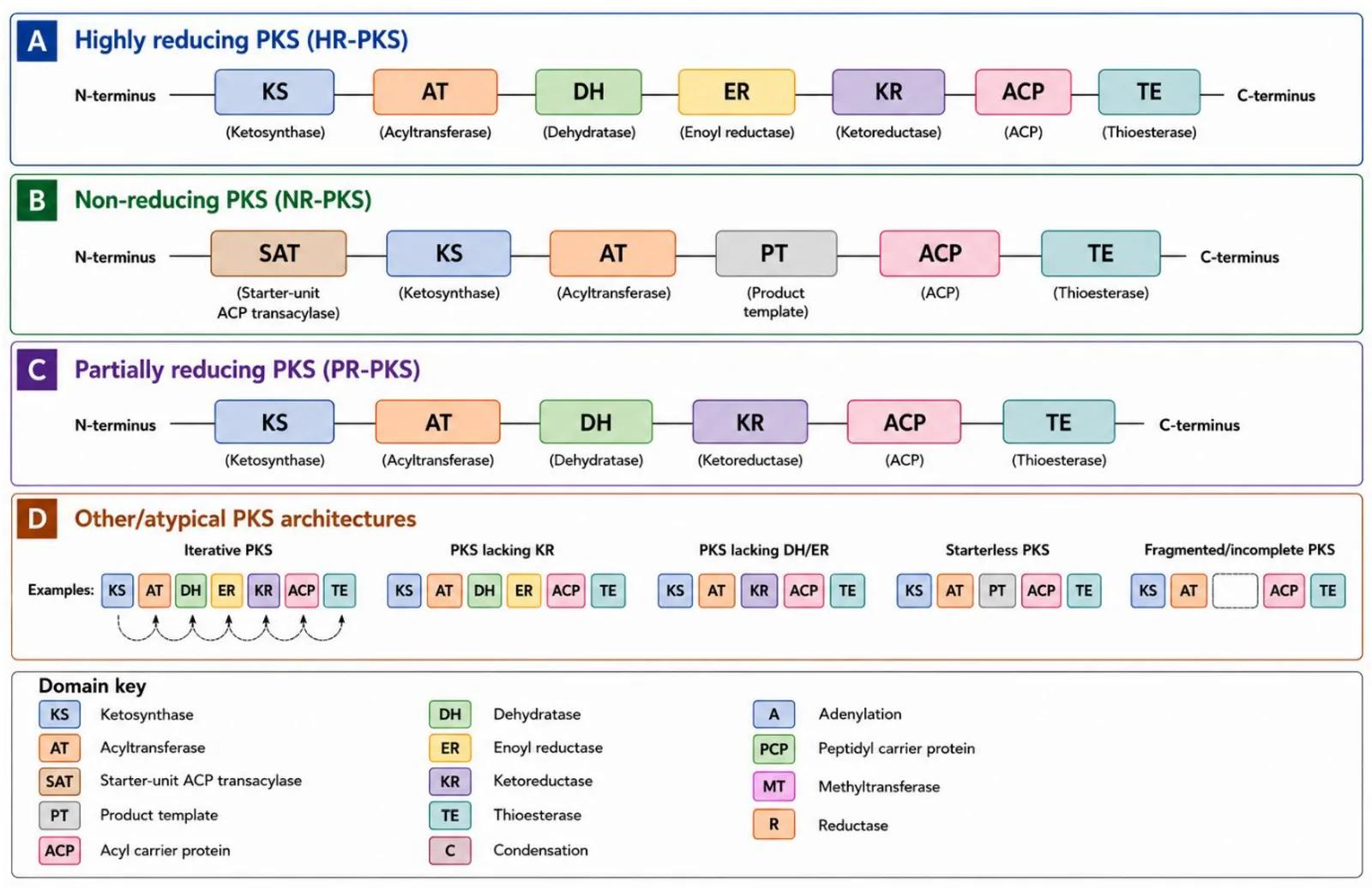
Representative domain architectures of polyketide synthases identified across 24 *Talaromyces* species. Schematic representation of the major non-hybrid PKS architectures: (**A**) highly reducing PKSs (HR-PKSs), (**B**) non-reducing PKSs (NR-PKSs), (**C**) partially reducing PKSs (PR-PKSs), and (**D**) atypical or non-canonical PKSs. Architectures are schematic and represent recurrent domain organizations identified in the analyzed PKS proteins.

After excluding the 86 fused PKS–NRPS proteins, 709 non-hybrid PKSs were subjected to domain-based classification. These comprised 323 highly reducing PKSs (HR-PKSs), 242 non-reducing PKSs (NR-PKSs), 92 partially reducing PKSs (PR-PKSs), seven type III PKSs (T3PKSs), and 45 atypical or non-canonical PKSs. HR-PKSs were the most abundant class and ranged from five in *T. trachyspermus* to 22 in *T. variabilis*. Their domain architectures included conserved ketosynthase (KS) and acyltransferase (AT) domains together with variable combinations of ketoreductase (KR), dehydratase (DH), and enoyl reductase (ER) domains (Figure 3A).

NR-PKS abundance ranged from two in *T. trachyspermus* to 16 each in *T. amestolkiae* and *T. fuscoviridis*. In addition to KS and AT domains, NR-PKS proteins frequently contained starter-unit acyltransferase (SAT) and/or product-template (PT) domains (Figure 3B). PR-PKSs occurred at lower abundance, with one to eight proteins per species, and displayed domain architectures with fewer reductive domains than HR-PKSs (Figure 3C). T3PKSs were detected as single representatives in *T. adpressus*, *T. borbonicus*, *T. nanjingensis*, *T. piceae*, *T. rugulosus*, *T. variabilis*, and *T. verruculosus*. A further 45 PKSs exhibited atypical or non-canonical domain organizations and could not be assigned unambiguously to the HR-PKS, NR-PKS, PR-PKS, or T3PKS classes (Figure 3D).

Comparison with characterized MIBiG pathways identified significant matches for 349 of the 667 PKS-associated BGCs (52.3%), corresponding to 102 distinct predicted metabolite associations. These included BGCs related to squalestatin S1, duclauxin, monorden/monocillins, pyrrocidine A/B, trypacidin, aurofusarin, sorbicillins, azanigerones, betaenones, and ankaflavin/monascin-related metabolites. The remaining 318 PKS-associated BGCs (47.7%) lacked significant MIBiG matches and were classified as chemically unresolved.

### 3.4. Comparative Analysis of NRPS-Containing Biosynthetic Gene Clusters and NRPS Domain Architecture

Of the 1550 predicted BGCs, 328 (21.2%) contained a canonical nonribosomal peptide synthetase (NRPS) component. The number of NRPS-containing BGCs ranged from 10 in *T. adpressus*, *T. liani*, *T. nanjingensis*, and *T. ruber* to 20 in *T. islandicus*. Relatively high numbers were also detected in *T. atroroseus*, *T. stollii*, and *T. variabilis* (17 BGCs each) and in *T. fuscoviridis*, *T. stipitatus*, and *T. wortmannii* (16 each) (Figure 2).

A total of 370 canonical NRPS core proteins were identified within these regions. NRPS protein abundance ranged from 10 in *T. adpressus* and *T. liani* to 31 in *T. atroroseus*, followed by 21 in *T. islandicus*. *T. funiculosus*, *T. fuscoviridis*, *T. stollii*, *T. trachyspermus*, and *T. variabilis* each contained 18 NRPS proteins (Table S1). In total, 36 NRPS-containing regions encoded multiple NRPS core genes, with up to four NRPS core genes detected within a single BGC. In *T. atroroseus*, 31 NRPS proteins were distributed among 17 NRPS-associated BGCs.

**Figure 4:**
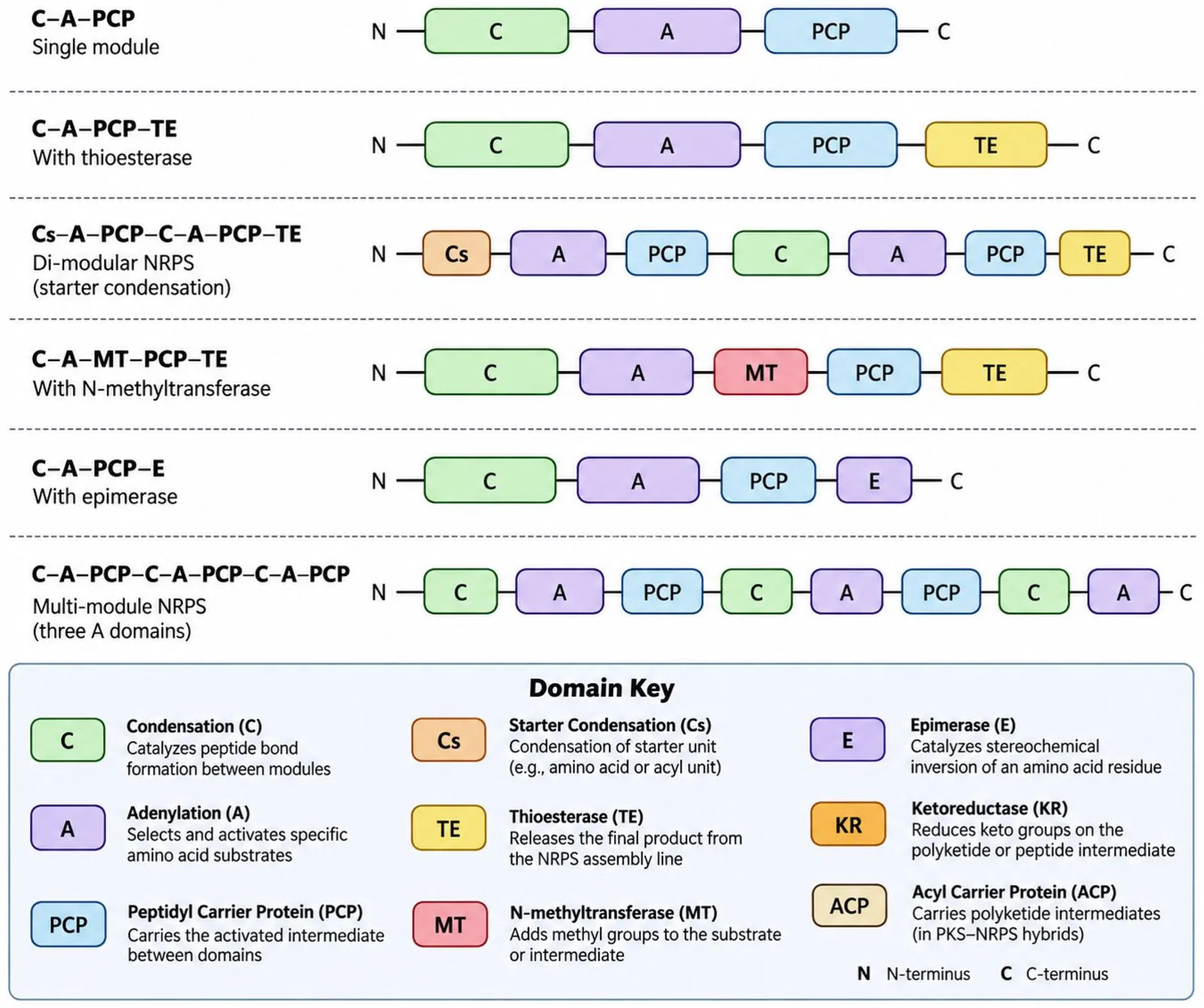
Domain organization and modular diversity of NRPS proteins across 24 *Talaromyces* species. Representative architectures illustrating variation in the number and arrangement of core adenylation (A), condensation (C), and peptidyl carrier protein (PCP) domains, together with accessory domains detected in the NRPS repertoire. The architectures illustrate the diversity of single-module and multimodular NRPS proteins identified across the genus and are schematic rather than drawn to scale.

Domain analysis showed that all 370 NRPS proteins contained detectable adenylation (A) and condensation (C) domains, while 346 (93.5%) also contained a detectable peptidyl carrier protein (PCP) domain. The remaining 24 proteins lacked a detectable PCP domain (Figure 4). Representative single-module and multimodular NRPS architectures are shown in Figure 4.

Comparison with characterized MIBiG pathways showed that 120 of the 328 NRPS-associated BGCs (36.6%) had significant matches, whereas 208 (63.4%) lacked a significant MIBiG match and were classified as chemically unresolved. Matched BGCs were associated with pathways related to burnettramic acid A, terrestric acid, emericellamides, leucinostatins, ilicicolin H, phyllostictines, chrodrimanin B, eupenifeldin, and sordarin.

### 3.5. Comparative Analysis of Terpene and Hybrid PKS–NRPS Biosynthetic Gene Clusters

Of the 1550 predicted BGCs, 345 (22.3%) contained a terpene-associated biosynthetic signature, whereas 126 (8.1%) contained both PKS and NRPS components and were classified as hybrid PKS–NRPS BGCs. Eight regions contained terpene, PKS, and NRPS signatures and were therefore included in both categories. After accounting for this overlap, terpene-associated and hybrid PKS–NRPS pathways comprised 463 non-redundant BGC regions, representing 29.9% of the total predicted BGC repertoire.

The number of terpene-associated BGCs ranged from four in *T. trachyspermus* to 23 in *T. verruculosus*, with a mean of 14.4 BGCs per genome. Relatively high numbers were also detected in *T. funiculosus* and *T. wortmannii* (20 BGCs each) and in *T. borbonicus* and *T. rugulosus* (19 each). The proportion of terpene-associated BGCs relative to the total BGC complement ranged from 9.8% in *T. trachyspermus* to 38.0% in *T. borbonicus* and 35.1% in *T. funiculosus*.

**Figure 5:**
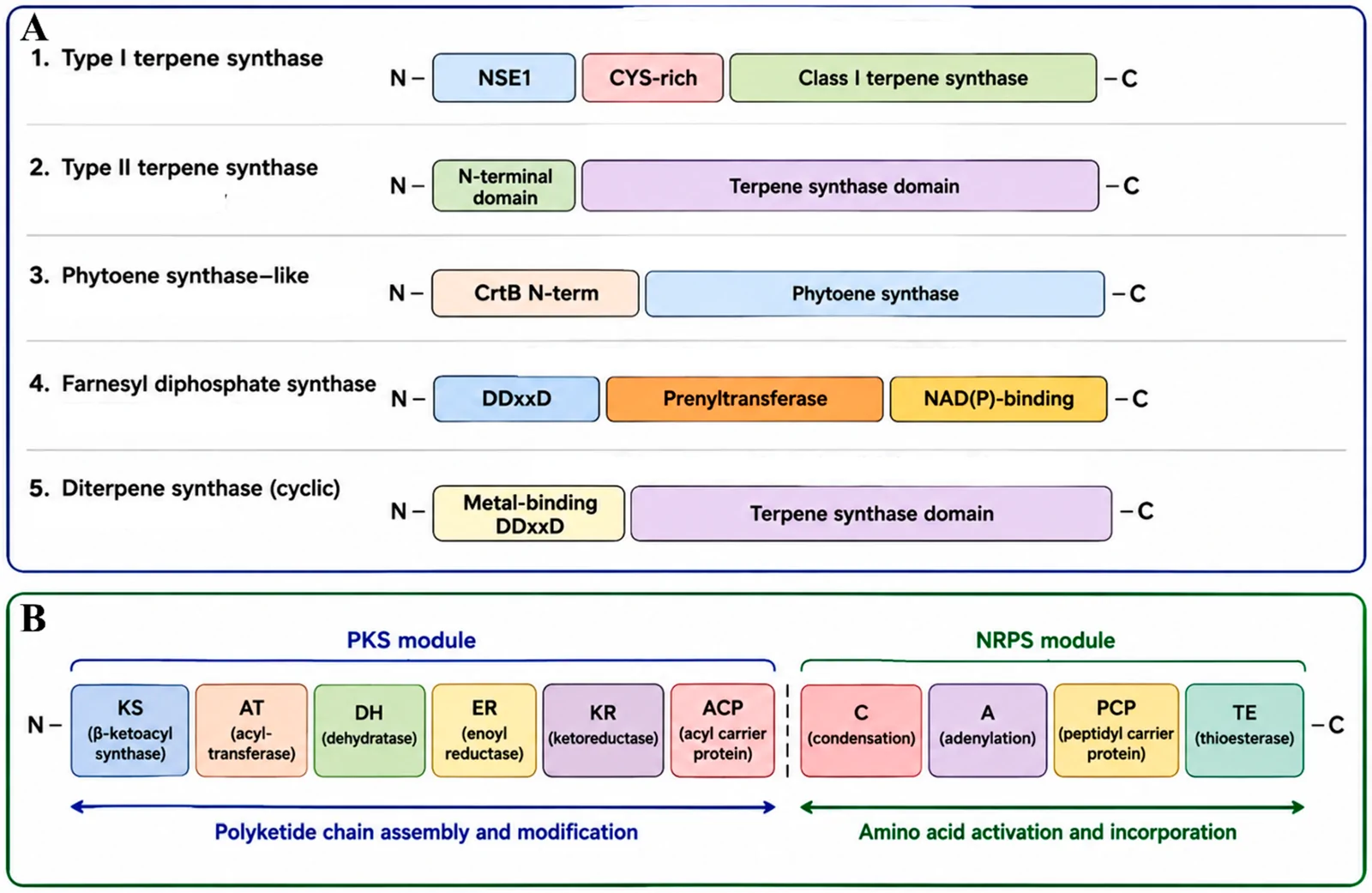
Representative domain architectures of terpene biosynthetic enzymes and hybrid PKS–NRPS proteins in *Talaromyces*. (**A**) Representative domain organizations of terpene-associated biosynthetic proteins identified across the 24 genomes, illustrating the major recurring terpene synthase and prenyltransferase architectures. (**B**) Representative architecture of a fused hybrid PKS–NRPS megasynthase showing the PKS module containing KS, AT, DH, ER, KR, and ACP domains and the NRPS module containing C, A, PCP, and TE domains. Architectures are schematic and are not drawn to scale.

Protein-level analysis identified 365 terpene-associated core biosynthetic proteins across the 24 genomes, ranging from four in *T. trachyspermus* to 24 each in *T. funiculosus* and *T. verruculosus*. Type I terpene synthase (T1TS)-associated proteins were the most abundant group, comprising 156 proteins (42.7%), followed by 83 UbiA-type prenyltransferases (22.7%), 72 type II terpene synthases (T2TSs; 19.7%), and 37 phytoene-synthase-like proteins (10.1%) (Figure 5A; Table S1). Seven proteins contained combined T1TS and T2TS signatures, six were classified as farnesyl diphosphate synthase (FPPS)-like prenyltransferases, and four contained Pyr4-like signatures. In addition, 55 regions were annotated by antiSMASH as terpene-precursor BGCs and were retained as a separate category from canonical terpene regions.

Hybrid PKS–NRPS BGCs were detected in all 24 *Talaromyces* genomes. Their abundance ranged from two in *T. liani* and *T. proteolyticus* to nine in *T. purpureogenus*, with a mean of 5.3 BGCs per genome. *T. islandicus* contained eight hybrid BGCs, whereas *T. funiculosus*, *T. fuscoviridis*, *T. stipitatus*, *T. trachyspermus*, and *T. verruculosus* each contained seven.

At the protein level, 86 fused PKS–NRPS proteins were identified. The highest number was detected in *T. atroroseus* (eight proteins), followed by *T. purpureogenus* (seven) and *T. fuscoviridis* and *T. wortmannii* (six each). Representative fused proteins contained PKS-associated ketosynthase (KS) and acyltransferase (AT) domains, frequently accompanied by dehydratase (DH), enoyl reductase (ER), and ketoreductase (KR) domains, together with NRPS-associated condensation (C), adenylation (A), and/or peptidyl carrier protein (PCP) domains (Figure 5B).

The difference between the 126 hybrid PKS–NRPS BGCs and 86 fused PKS–NRPS proteins resulted from the use of region-level and protein-level classifications, respectively. Hybrid BGCs included regions containing both PKS and NRPS components, irrespective of whether these components were encoded by a single fused protein or by separate proteins within the same predicted cluster.

MIBiG comparison identified terpene-associated BGCs with similarity to characterized pathways for clavaric acid, squalestatin S1, asperterpenoid A, ochrindole A, brassicicene C, gibberellins, aphidicolin-related metabolites, terpestacin, and sordarin-related systems. Hybrid PKS–NRPS BGCs showed similarity to characterized pathways associated with burnettramic acid A, terrestric acid, emericellamides, leucinostatins, ilicicolin H, phyllostictines, and chrodrimanin B.

### 3.6. Distribution of Minor and Specialized Biosynthetic Gene Cluster Classes

**Figure 6:**
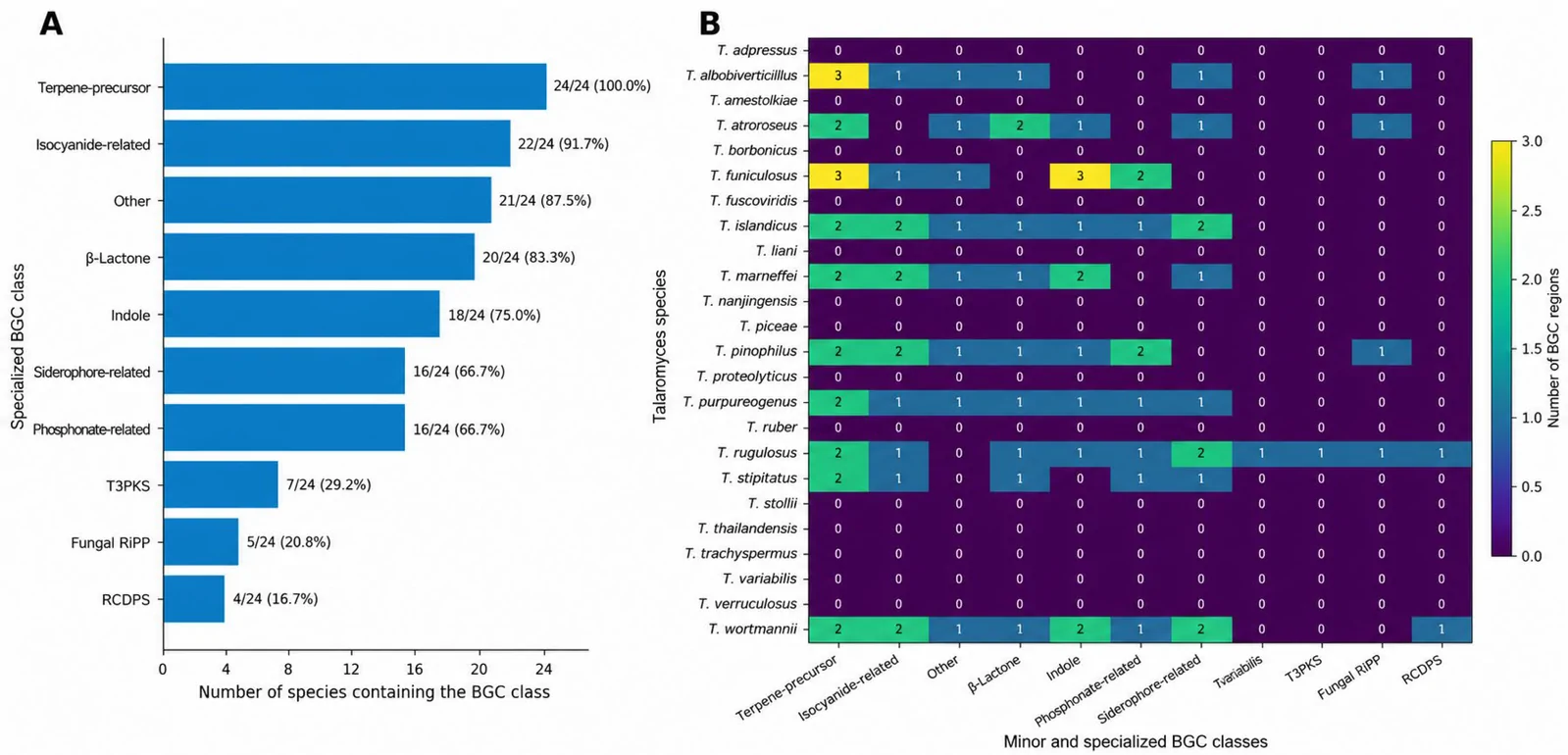
Distribution of minor and specialized biosynthetic gene cluster (BGC) classes across 24 *Talaromyces* species. (**A**) Number and proportion of species containing each specialized BGC class. Values beside the bars indicate the number of species in which each class was detected and the corresponding percentage of the 24 species analyzed. (**B**) Heatmap showing the species-level distribution and abundance of these BGC classes, with cell values representing the number of BGC regions detected in each species. Related antiSMASH annotations were grouped into broader categories, including isocyanide-related, phosphonate-related, and siderophore-related BGCs. T3PKS, and fungal RiPP, represent comparatively rare classes. Because individual antiSMASH regions may contain multiple biosynthetic signatures, the displayed class counts represent non-exclusive BGC annotations.

In addition to PKS-, NRPS-, terpene-, and hybrid-associated pathways, the 24 *Talaromyces* genomes contained several less abundant biosynthetic gene cluster (BGC) classes, including isocyanide and isocyanide–nonribosomal peptide (isocyanide-NRP), indole, phosphonate and phosphonate-like, nonribosomal peptide (NRP)-metallophore and non-integrated (NI)-siderophore, β-lactone, and fungal ribosomally synthesized and post-translationally modified peptide (RiPP) clusters (Figure 6). Because individual antiSMASH regions could receive more than one product-type annotation, these categories were non-exclusive and were not summed as independent BGC totals.

A total of 38 isocyanide-associated BGC annotations were detected across 22 of the 24 species, comprising 21 canonical isocyanide and 17 isocyanide-NRP regions [31]. The number of isocyanide-associated regions ranged from zero to three per genome, with the highest numbers detected in *T. amestolkiae*, *T. nanjingensis*, and *T. stollii*. No isocyanide-associated BGCs were detected in *T. atroroseus* or *T. trachyspermus*. Several isocyanide-associated regions also contained PKS-, NRPS-, or terpene-associated product-type annotations.

Indole-associated BGCs comprised 33 regions distributed across 18 species, with zero to four regions detected per genome [32]. *T. verruculosus* contained the highest number (four regions), whereas *T. adpressus*, *T. funiculosus*, and *T. variabilis* each contained three. Phosphonate-associated BGCs comprised 25 regions across 16 species, including 16 canonical phosphonate and nine phosphonate-like regions [33]. Some phosphonate-associated regions also carried additional product-type annotations. One terpene-precursor/phosphonate region showed similarity to the characterized fosfonochlorin biosynthetic system.

Siderophore-associated pathways comprised 22 BGCs across 16 species, including 13 NRP-metallophore and nine NI-siderophore regions [34]. The highest combined numbers of these regions were detected in *T. islandicus*, *T. liani*, *T. proteolyticus*, *T. rugulosus*, *T. variabilis*, and *T. wortmannii*. NRP-metallophore regions frequently overlapped with NRPS-associated annotations. In *T. rugulosus*, one such region contained an ornithine monooxygenase together with a multidomain NRPS core enzyme.

β-Lactone BGCs were detected in 20 species, with 22 regions identified across the dataset [35]. Most genomes contained a single β-lactone region, whereas *T. atroroseus* and *T. proteolyticus* each contained two. Fungal RiPP-associated BGCs were detected in five species, with one region each in *T. albobiverticillius*, *T. atroroseus*, *T. pinophilus*, *T. rugulosus*, and *T. variabilis* [36]. The RiPP-associated region in *T. rugulosus* encoded proteins containing Peptidase_S41 and DUF3328 domains and showed similarity to characterized pathways associated with phomopsins and ustiloxin B.

### 3.7. Conservation and Diversification of Biosynthetic Gene Cluster Families Across Talaromyces

Analysis of the 1550 predicted BGCs across 24 *Talaromyces* species resolved them into 828 gene cluster families (GCFs) (Table S2). GCF distribution was strongly skewed toward narrowly distributed families. Of these, 567 GCFs (68.5%) were species-specific, 120 were shared by two species, and 58 were shared by three species. Thus, 745 of the 828 GCFs (90.0%) occurred in three or fewer species, whereas only 83 were detected in four or more species. No individual GCF was conserved across all 24 genomes. The most widely distributed family, GCF523, occurred in 23 species, followed by GCF79 in 17 species, GCF60 and GCF73 in 15 species each, and GCF152, GCF711, GCF763, and GCF769 in 12 species each.

**Figure 7:**
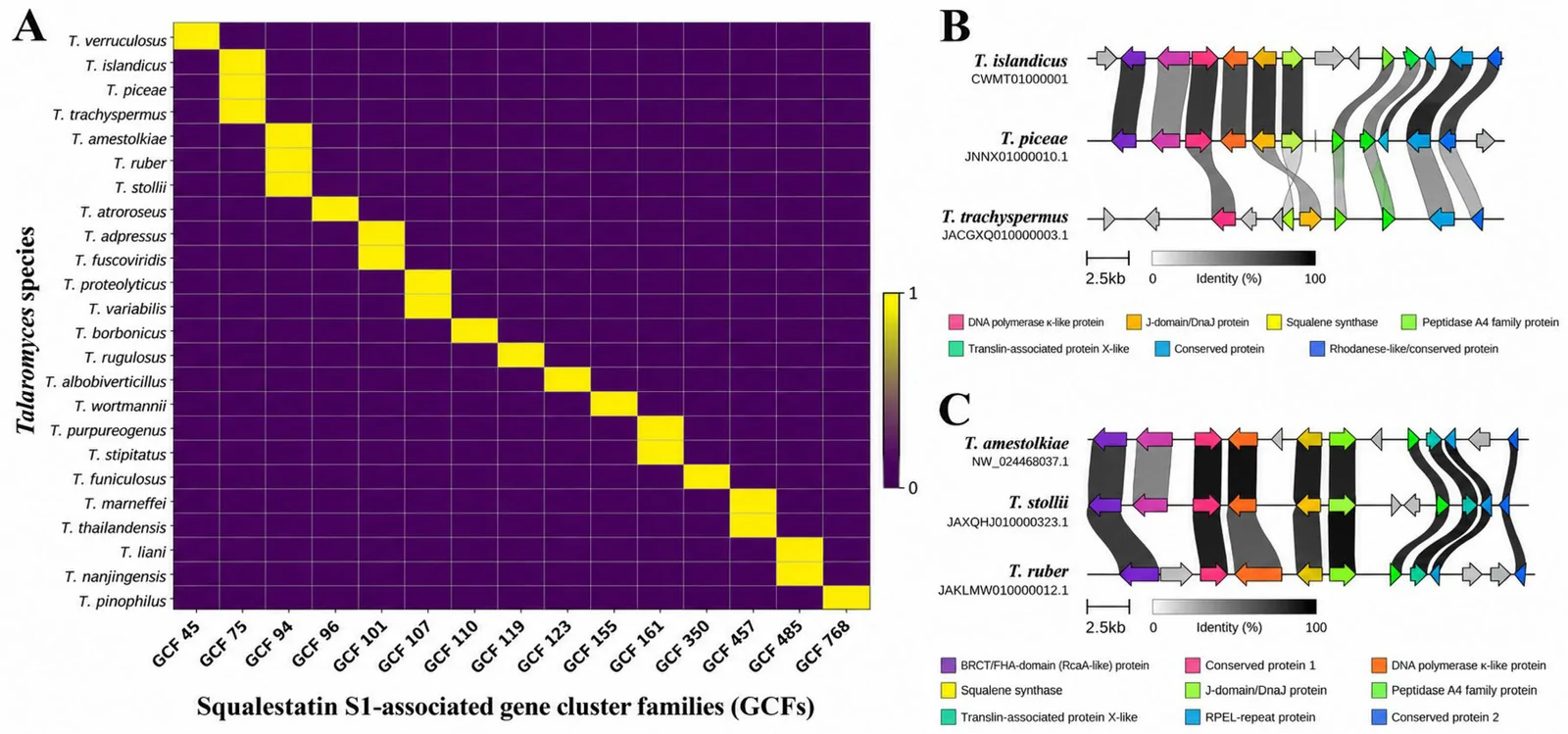
Conservation and structural diversification of squalestatin S1-associated biosynthetic gene clusters across *Talaromyces.* (**A**) Distribution of squalestatin S1-associated BGCs among the 24 species and their assignment to distinct BiG-SLiCE gene cluster families (GCFs). (**B**) Comparative organization of GCF75 BGCs from *T. islandicus*, *T. piceae*, and *T. trachyspermus*, highlighting seven conserved homologous genes. (**C**) Comparative organization of GCF94 BGCs from *T. amestolkiae*, *T. stollii*, and *T. ruber*, highlighting nine conserved homologous genes. Conservation of the squalene synthase gene across these clusters indicates retention of a key biosynthetic component despite variation in surrounding cluster architecture. Comparative BGC organization was visualized using clinker, with homologous genes represented by corresponding colors and similarity links indicating protein sequence similarity between cluster.

Despite the absence of a single GCF conserved across all species, BGCs showing similarity to the characterized squalestatin S1 pathway (MIBiG BGC0001839.3) were detected in all 24 genomes [37]. These BGCs were distributed among 15 GCFs (GCF45, GCF75, GCF94, GCF96, GCF101, GCF107, GCF110, GCF119, GCF123, GCF155, GCF161, GCF350, GCF457, GCF485, and GCF768), demonstrating broad conservation of a squalestatin-associated biosynthetic signature despite substantial variation in cluster architecture (Figure 7A). This structural diversification was illustrated by GCF75 and GCF94. GCF75, shared by *T. islandicus*, *T. piceae*, and *T. trachyspermus*, contained seven conserved homologous genes, whereas GCF94, shared by *T. amestolkiae*, *T. stollii*, and *T. ruber*, contained nine (Figure 7B,C). Both GCFs contained a homologous squalene synthase-associated biosynthetic component, while differences were observed in the number, identity, and organization of surrounding genes.

Clavaric acid-associated BGCs were also detected across all 24 species but were distributed among four GCFs. Among these, GCF73 was the most widely distributed, occurring in 15 species, with 14 constituent BGCs showing the same MIBiG association. Other broadly distributed metabolite-associated families included GCF769, which occurred in 12 species and was associated with the ankaflavin/monascin/rubropunctatine/monascorubrin biosynthetic system, and GCF711, also present in 12 species and predominantly associated with azanigerones A–F.

GCF523, the most widely distributed individual family, was detected in 23 species and was absent only from *T. trachyspermus* (Figure S1). All 23 constituent BGCs showed significant MIBiG matches, with 15 most similar to the aurofusarin pathway (BGC0002709.2) and eight to the YWA1 pathway (BGC0002175.3). Of the 23 GCF523 members, 22 were classified as PKS-containing BGCs, whereas one exhibited a hybrid PKS–NRPS architecture. Comparative organization of the GCF523 members across the 23 species is shown in Figure S1.

**Figure 8:**
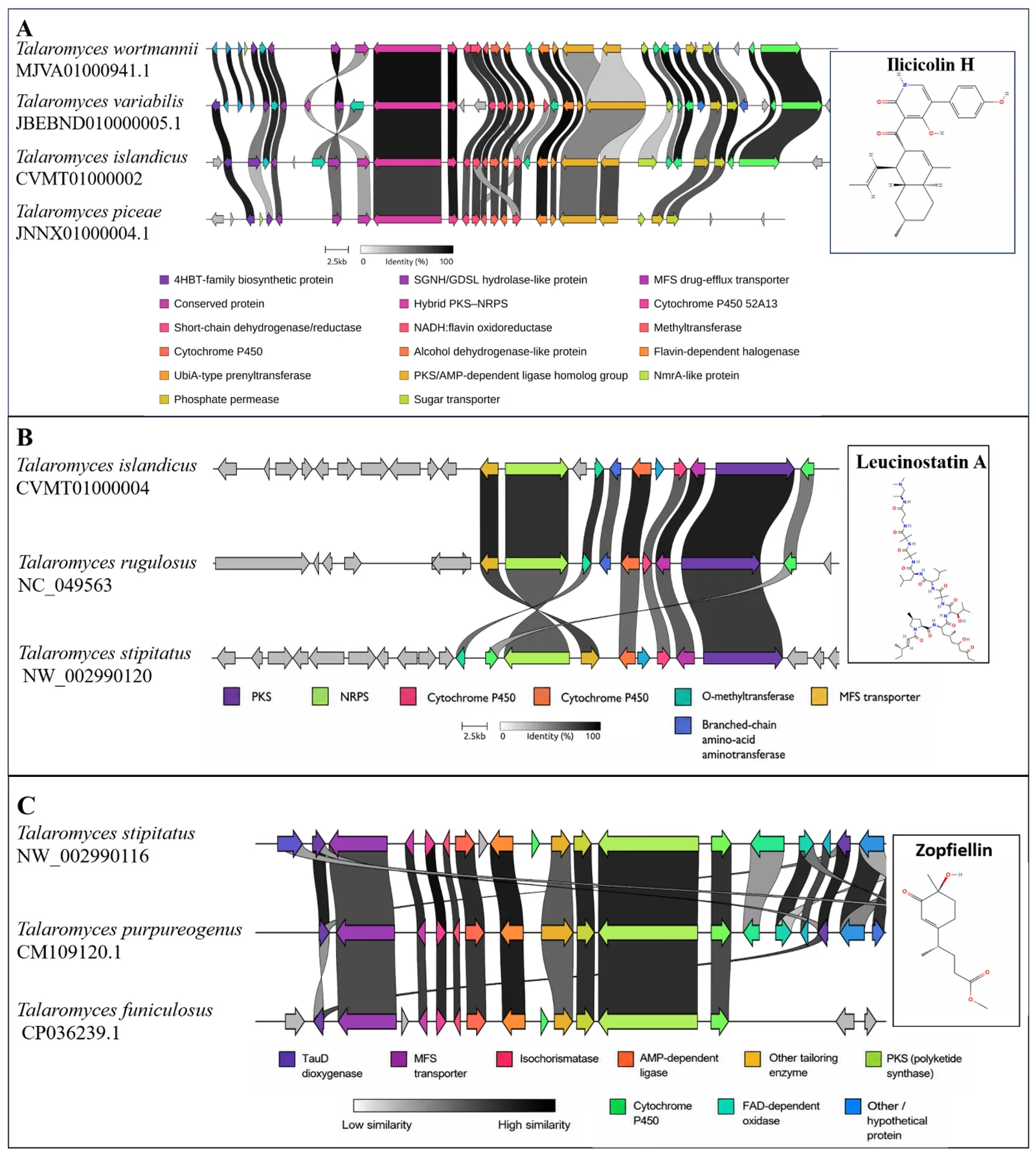
Comparative organization of antifungal metabolite-associated gene cluster families in *Talaromyces*. Representative multi-species GCFs associated with (**A**) ilicicolin H (GCF822), (**B**) leucinostatin A (GCF659), and (**C**) zopfiellin (GCF685). Homologous genes are indicated by corresponding colors, illustrating conservation and variation in gene content and organization among species. Metabolite assignments are based on similarity to characterized MIBiG BGCs and represent predicted biosynthetic associations rather than experimentally confirmed metabolite production. Comparative BGC organization was visualized using clinker, with homologous genes represented by corresponding colors and similarity links indicating protein sequence similarity between cluster.

Several metabolite-associated GCFs showed more restricted distributions across the analyzed species (Figure 8). Ilicicolin H-associated GCF822 was detected in *T. islandicus*, *T. piceae*, *T. variabilis*, and *T. wortmannii*. Leucinostatin A-associated GCF659 occurred in *T. islandicus*, *T. rugulosus*, and *T. stipitatus*, whereas zopfiellin-associated GCF685 was detected in *T. purpureogenus*, *T. stipitatus*, and *T. verruculosus* (Figure 8).

Several more restricted GCFs occurred in limited species. For example, Ilicicolin H-associated GCF822 occurred in *T. islandicus*, *T. piceae*, *T. variabilis*, and *T. wortmannii*; leucinostatin A-associated GCF659 occurred in *T. islandicus*, *T. rugulosus*, and *T. stipitatus*; and zopfiellin-associated GCF685 was detected in *T. purpureogenus*, *T. stipitatus*, and *T. verruculosus* (Figure 8). Overall, the GCF analysis revealed extensive lineage-level diversification alongside selective conservation of particular biosynthetic systems. Most GCFs were restricted to three or fewer species, whereas several metabolite-associated pathways were broadly distributed across the genus. Particularly notable was the occurrence of squalestatin S1-associated BGCs in all 24 species despite their distribution among 15 distinct GCFs, demonstrating that conservation of predicted biosynthetic capacity can coexist with substantial structural diversification of the underlying clusters.

### 3.8. Lineage-Specific and Chemically Unresolved Biosynthetic Potential

In addition to broadly conserved biosynthetic systems, the analysis revealed extensive lineage-specific variation across *Talaromyces*. Among the species-specific GCFs, 168 showed similarity to characterized metabolite-associated pathways, predominantly involving PKS, NRPS, hybrid PKS–NRPS, and terpene biosynthesis. Their abundance varied considerably among species, with the largest numbers detected in *T. proteolyticus* (17), T. atroroseus (16), *T. borbonicus* (15), and *T. albobiverticillius* (14), indicating substantial differences in lineage-specific biosynthetic repertoires.

**Figure 9:**
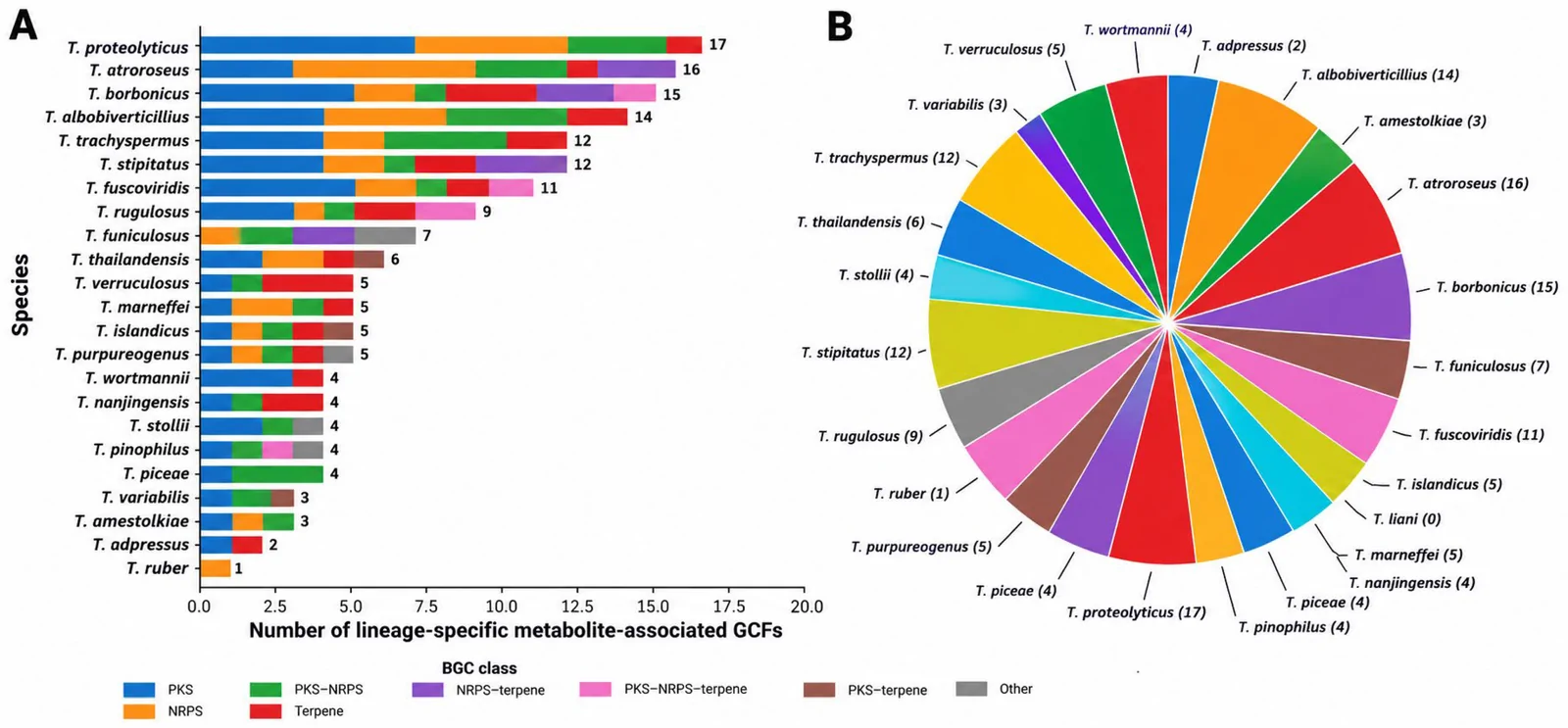
Lineage-specific and chemically unresolved biosynthetic diversity across 24 *Talaromyces* species. (**A**) Distribution of species-specific GCFs associated with characterized secondary metabolites, illustrating interspecific differences in lineage-restricted biosynthetic potential. Representative lineage-specific metabolite associations include sordarin in *T. thailandensis* (GCF187), pyrrocidine A/B in *T. atroroseus* (GCF714), monorden/monocillins in *T. proteolyticus* (GCF810), and trypacidin in *T. pinophilus* (GCF516). (**B**) Distribution of GCFs lacking significant MIBiG matches across the 24 species, representing chemically unresolved biosynthetic families. Absence of a significant MIBiG match indicates lack of similarity to currently characterized BGCs and should not be interpreted as direct evidence of chemical novelty.

Several species-specific GCFs were associated with characterized pathways of potential biological relevance. These included a sordarin-associated GCF in *T. thailandensis* (GCF187), a pyrrocidine A/B-associated GCF in *T. atroroseus* (GCF714), a monorden/monocillin-associated GCF in *T. proteolyticus* (GCF810), and a trypacidin-associated GCF in *T. pinophilus* (GCF516) (Figure 9A).

A larger proportion of GCF diversity remained chemically unresolved. Of the 828 GCFs identified across the 24 genomes, 553 (66.8%) lacked a significant match to a characterized MIBiG cluster. Chemically unresolved GCFs occurred in every species but varied in abundance, ranging from 24 in *T. trachyspermus* to 56 in *T. variabilis*. Large complements were also detected in *T. wortmannii* (53), *T. pinophilus* (52), *T. adpressus* (50), and *T. rugulosus* and *T. verruculosus* (49 each) (Figure 9B).

## 4. Discussion

Comparative analysis of 24 *Talaromyces* species revealed a marked contrast between conservation of the broader gene repertoire and diversification of secondary-metabolite biosynthesis. Although most proteins were assigned to shared orthogroups, BGC abundance and composition varied substantially among species, indicating that specialized metabolism is a dynamic component of genome evolution. Fungal BGCs can diversify through gene gain and loss, duplication, rearrangement, divergence of biosynthetic enzymes, and horizontal transfer [15,38,39,40], and comparable patterns have been reported in other filamentous fungi [38,41]. Thus, the observed variation indicates extensive remodeling of secondary-metabolite capacity within an otherwise relatively conserved *Talaromyces* genomic framework.

### 4.1. Diversification of Major Biosynthetic Systems in Talaromyces

PKS-associated pathways constituted the largest component of the biosynthetic repertoire, consistent with the prominent contribution of polyketides to fungal chemical diversity and the known capacity of *Talaromyces* species to produce polyketides, azaphilones, quinones, and related metabolites [16]. Interspecific differences involved not only PKS abundance but also highly reducing, non-reducing, partially reducing, type III, and atypical architectures. Variation in catalytic and modifying domains can affect starter-unit selection, reduction state, cyclization, and final scaffold formation [42,43], providing a mechanistic basis for diversification of polyketide biosynthesis across the genus.

Several PKS-associated clusters were related to characterized pathways, including those associated with ankaflavin and monascin. These azaphilone metabolites have been investigated for diverse biological properties and potential biotechnological applications [44]. Their occurrence in multiple *Talaromyces* GCFs illustrates the diversity of pigment- and polyketide-associated pathways represented in the genus. A substantial proportion of PKS-associated BGCs nevertheless lacked significant MIBiG matches.

NRPS-associated pathways represented another important component of the biosynthetic repertoire. Variation in adenylation, carrier, and condensation-domain organization can alter substrate selection and peptide assembly [45]. The identified pathways included associations with ilicicolin H, leucinostatins, sordarin, burnettramic acid, terrestric acid, emericellamides, phyllostictines, chrodrimanin B, and eupenifeldin, illustrating the broad peptide-biosynthetic capacity of *Talaromyces*.

Terpene-associated pathways were also widespread. Structural diversity in fungal terpenes arises from cyclization of prenyl diphosphate precursors followed by pathway-specific enzymatic modifications [46]. The repertoire included an aphidicolin-related pathway in *T. nanjingensis*. Aphidicolin inhibits replicative eukaryotic DNA polymerases and has been widely used in studies of DNA replication [47], demonstrating the biological relevance of some terpene-associated biosynthetic signatures identified here.

Hybrid PKS–NRPS pathways further expand the potential chemical repertoire by integrating polyketide and nonribosomal-peptide biosynthetic machinery. Such systems are established sources of structurally complex fungal natural products [48]. Their occurrence across the analyzed species, as both fused megasynthases and clusters containing separate PKS and NRPS components, demonstrates substantial flexibility in the organization of hybrid biosynthetic systems.

### 4.2. Less Abundant Pathways Broaden the Metabolic Repertoire

Isocyanide, indole, phosphonate, siderophore, β-lactone, and fungal RiPP pathways contributed additional biosynthetic capacity beyond the dominant PKS, NRPS, and terpene systems. Their uneven distribution among species indicates that differences in *Talaromyces* secondary metabolism extend across multiple biosynthetic classes.

Siderophore-associated pathways are particularly relevant to fungal ecology because siderophores mediate high-affinity iron acquisition and can influence competition among microorganisms [49]. Their occurrence in multiple *Talaromyces* species suggests roles in iron homeostasis and competitive interactions, processes that may also contribute to the ecological performance of strains with biocontrol potential.

Fungal RiPP-associated regions were comparatively restricted. Unlike PKS- and NRPS-derived metabolites, RiPPs originate from ribosomally synthesized precursor peptides that subsequently undergo enzymatic modification. The identification of regions related to phomopsin- and ustiloxin-type systems [50,51] therefore adds a distinct biosynthetic mechanism to the metabolic repertoire of the genus and highlights these less abundant pathways as candidates for further characterization.

### 4.3. Conserved Biosynthetic Capacity Despite Structural Remodeling

The GCF analysis showed that conservation of related biosynthetic capacity does not necessarily require identical cluster architecture. Most GCFs were narrowly distributed despite extensive conservation of the broader orthologue repertoire, consistent with the evolutionary plasticity of fungal BGCs [15,38]. Similar remodeling of cluster composition has been documented in other fungal secondary-metabolite systems [52].

The squalestatin S1-associated pathways provide a clear example. Related BGCs were detected throughout the genus but were distributed among multiple GCFs, indicating substantial variation in cluster organization despite retention of a common biosynthetic association. This pattern is particularly notable because squalestatin S1 is a potent inhibitor of squalene synthase [53].

Clavaric acid-associated pathways showed a comparable distribution, occurring in several GCFs and broadly across the analyzed species. Clavaric acid inhibits farnesyl-protein transferase [54], and related analogues have also been investigated as inhibitors of Ras-associated protein prenylation [55]. Its distribution across several GCFs provides another example of this pattern.

GCF523 represents a different pattern, being retained in nearly all analyzed species and associated with aromatic-polyketide biosynthesis. Its similarity to both aurofusarin- and YWA1-related systems is more appropriately interpreted as conservation of related biosynthetic features than as evidence for production of a single common metabolite, because variation in downstream tailoring reactions can redirect related intermediates toward different products.

Other characterized pathways were retained only in subsets of the genus, including those related to ilicicolin H, leucinostatins, and zopfiellin. Ilicicolin H inhibits fungal mitochondrial respiration through the cytochrome bc1 complex [56], whereas zopfiellin has demonstrated antifungal activity against phytopathogenic *Colletotrichum* and *Botrytis* species [57]. These pathways therefore show markedly different patterns of conservation across the genus.

### 4.4. Lineage-Specific Pathways and Unexplored Biosynthetic Potential

The predominance of species-specific GCFs indicates extensive species-level differentiation of secondary-metabolite capacity. Because fungal secondary metabolites frequently mediate interactions with hosts, competitors, and environmental substrates, such restricted pathways may contribute to ecological specialization [38,46]. Importantly, several of these GCFs were related to experimentally characterized metabolites, showing that species-level differentiation is not confined to chemically unresolved clusters.

The sordarin-associated pathway is particularly informative. Sordarins inhibit fungal protein synthesis by interfering with elongation-factor-dependent ribosomal translocation [58,59], and a sordarin biosynthetic cluster has been experimentally characterized in *T. adpressus* [60]. This provides direct precedent for translating *Talaromyces* genome-mining predictions into experimentally validated metabolite pathways. Similarly, the monorden/monocillin association is agriculturally relevant because monorden has been investigated as a fungicidal lead against phytopathogenic fungi [61].

Other restricted associations broaden the potential functional significance of the dataset. A griseofulvin/epidechlorogriseofulvin-related PKS pathway was restricted to *T. wortmannii*, and griseofulvin derivatives have shown antifungal activity against phytopathogenic fungi [62]. A mycophenolic-acid-associated PKS pathway was identified in *T. stipitatus*; mycophenolic acid derivatives are well known for immunosuppressive activity [63]. These findings illustrate how differences in BGC repertoires among species can encompass pathways associated with distinct biological activities.

A pyripyropene A-associated GCF was identified in a small subset of species. Pyripyropene chemistry is agriculturally relevant because it provided the chemical basis for development of the insecticide afidopyropen [64]. However, this GCF showed mixed MIBiG assignments in the supplementary analysis, and the pyripyropene association should therefore be regarded as tentative.

Finally, the large proportion of GCFs lacking significant MIBiG matches indicates that much of the *Talaromyces* biosynthetic repertoire remains chemically unresolved. Such clusters should not automatically be considered novel, because the absence of a characterized match may reflect pathway divergence, incomplete linkage between known metabolites and their BGCs, or limitations in genome annotation and available reference databases. Candidate pathways can therefore be prioritized on the basis of unusual core-enzyme architecture, distinctive tailoring-gene combinations, phylogenetic distribution, and relationships to biologically relevant characterized pathways. Experimental characterization of the sordarin cluster [60] and activation of a silent *Talaromyces* pathway yielding cryptic azaphilones [19] demonstrate the feasibility of genome-guided prioritization. Transcriptomic, metabolomic, and functional analyses will ultimately be required to establish pathway activity, metabolite production, and biological function. Overall, the coexistence of broadly conserved biosynthetic systems, extensive GCF diversification, lineage-specific characterized pathways, and a large chemically unresolved repertoire highlights *Talaromyces* as a metabolically diverse genus with substantial potential for genome-guided discovery of biologically relevant secondary metabolites.

## 5. Conclusions

Comparative genomic analysis of 24 *Talaromyces* species revealed extensive diversification of secondary-metabolite biosynthesis despite substantial conservation of the overall genomic repertoire. The 1550 predicted BGCs were grouped into 828 GCFs, of which 68.5% were species-specific, demonstrating pronounced lineage-level diversification. In parallel, several broadly conserved and multi-species GCFs were associated with characterized biosynthetic pathways, indicating selective conservation of particular metabolic capacities. The occurrence of antifungal metabolite-associated pathways, together with a large chemically unresolved GCF repertoire, highlights *Talaromyces* as a promising resource for genome-guided natural-product discovery and biocontrol research. These genomic predictions provide candidates for prioritization but require metabolomic, transcriptional, and functional validation to confirm metabolite production and biological activity.

## Figures and Tables

**Table 1:** Genome and orthologue statistics of 24 *Talaromyces* species used in this study.

Species	Assembly Accession	Genome Size (Mb)	Total Proteins	Orthogroups	Singletons/ Unassigned Proteins
*T. adpressus*	GCA_002775195.1	36.1	11,064	6790	76
*T. albobiverticillius*	GCA_023721895.2	38.6	12,227	6751	250
*T. amestolkiae*	GCF_001896365.1	33.7	10,671	6709	59
*T. atroroseus*	GCA_051125915.1	30.4	13,678	6747	288
*T. borbonicus*	GCA_002916415.1	27.1	8100	5964	94
*T. funiculosus*	GCA_004299765.1	28.5	9101	6318	67
*T. fuscoviridis*	GCA_059714275.1	36.9	11,163	6789	104
*T. islandicus*	GCA_000985935.1	34.7	9927	6637	112
*T. liani*	GCA_022814505.1	35.5	10,696	6771	72
*T. marneffei*	GCF_009556855.1	28.6	9994	6559	137
*T. nanjingensis*	GCA_031010415.1	42.5	11,612	6865	102
*T. piceae*	GCA_001657655.1	26.6	8100	6016	97
*T. pinophilus*	GCA_001571465.2	36.5	11,292	6849	49
*T. proteolyticus*	GCA_055862385.1	37.8	10,299	6360	191
*T. purpureogenus*	GCA_048569275.1	30.3	8991	6242	84
*T. ruber*	GCA_022813215.1	30	8844	6182	41
*T. rugulosus*	GCF_013368755.1	35.8	11,901	6990	274
*T. stipitatus*	GCF_000003125.1	35.7	13,250	6798	321
*T. stollii*	GCA_037040825.1	34.3	9959	6401	51
*T. thailandensis*	GCA_019828575.1	31.8	8876	6308	45
*T. trachyspermus*	GCA_020137715.1	32	8695	5434	384
*T. variabilis*	GCA_040333175.1	33.8	10,755	6666	83
*T. verruculosus*	GCA_038993655.2	40.6	11,227	6723	62
*T. wortmannii*	GCA_001939245.1	38.6	11,418	6903	156

## Data Availability

The original contributions presented in this study are included in the article/Supplementary Material. Further inquiries can be directed to the corresponding author.

## Supplementary Materials

The following supporting information can be downloaded at https://www.mdpi.com/article/10.3390/jof12090690/s1, Figure S1: Comparative organization of GCF523 BGCs associated with the aurofusarin biosynthetic pathway across *Talaromyces* species; Table S1: Distribution of core biosynthetic proteins associated with major biosynthetic gene cluster (BGC) classes across 24 *Talaromyces* species; Table S2: Details of GCF identified in 24 *Talaromyces* species.
