# Supplementary material for: Comparative Genomics Reveals Extensive Biosynthetic Diversity and Lineage-Specific Metabolic Potential Across Talaromyces Species
Source: J Fungi (Basel). 2026 Sep 14;12(9):690. doi: 10.3390/jof12090690 (PMC13608686; doi:10.3390/jof12090690)
Supplement: Supplementary file 1 [file jof-12-00690-s001.zip › Supplementary Figure S1.pdf]

Supplementary Figure

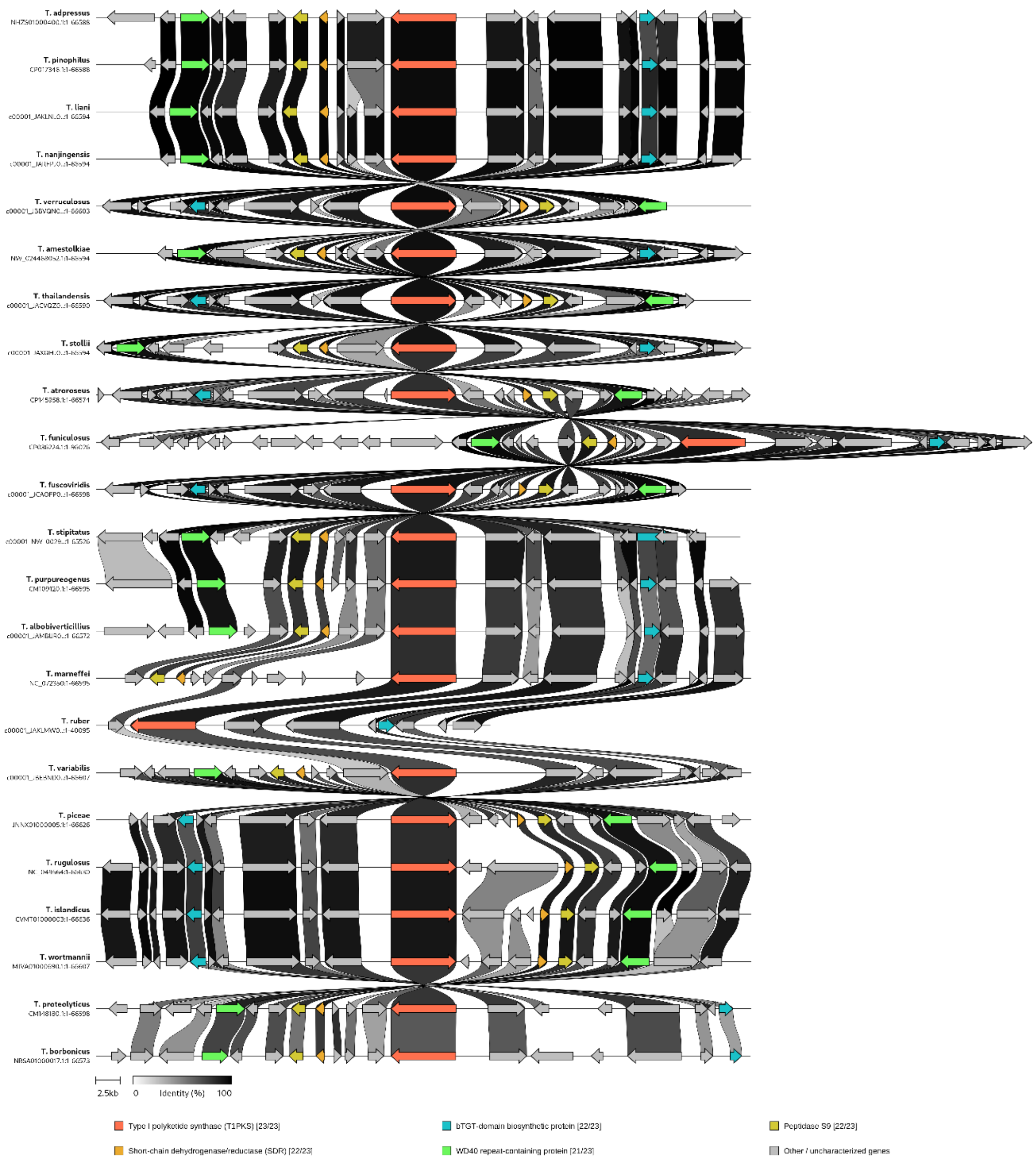

Figure S1. Comparative organization of GCF523 BGCs associated with the aurofusarin biosynthetic pathway across *Talaromyces* species. Gene-cluster organization and synteny of BGCs assigned to **GCF523** are shown in comparison with the characterized **aurofusarin biosynthetic gene cluster (MIBiG BGC0002709.2)**. Arrows represent predicted genes and their transcriptional orientation, with colors indicating different biosynthetic or functional gene categories. Grey connecting regions indicate homologous sequences between clusters and illustrate conservation of gene content and organization. Variation in gene presence, orientation, and cluster architecture indicates structural diversification of the aurofusarin-associated GCF across *Talaromyces* species.
